# Evaluating the real-world robustness of face-swap detection models under compression and noise

**DOI:** 10.3389/frai.2026.1835651

**Published:** 2026-06-23

**Authors:** Baocai Li, Fazli Bin Azzali, Nik Fatinah binti N. Mohd Farid

**Affiliations:** School of Computing, Universiti Utara Malaysia, Sintok, Malaysia

**Keywords:** compression artifacts, Deepfake, face-swap detection, FSD-GAN, media distortion, real-world evaluation, robustness, XceptionNet

## Abstract

**Introduction:**

Recent advances in generative adversarial networks (GANs) and autoencoding techniques have significantly improved the realism of face-swap and deepfake media, creating substantial challenges for digital media authentication. Although existing deepfake detection models achieve high accuracy on benchmark datasets, their robustness under real-world media degradations remains insufficiently explored.

**Methods:**

This study systematically evaluates the resilience of five leading face-swap detection models—XceptionNet, MesoNet, FSD-GAN, FakeTracer, and a Hybrid + Landmark approach—under four common distortions: JPEG compression (quality levels 20–90), Gaussian noise (*σ* = 0.01–0.05), motion blur (kernel size 3–15), and video encoding artefacts (bitrate 50–500 kbps). Experiments were conducted using the FaceForensics++ dataset (1,000 videos: 720 training, 140 validation, and 140 testing) and Celeb-DF v2 (590 videos: 400 training, 90 validation, and 100 testing). Performance was assessed using accuracy, F1-score, area under the curve (AUC), and degradation rate (*Δ*) between clean and distorted conditions.

**Results:**

The results demonstrate a substantial reduction in detection performance under degraded conditions. Average accuracy declined from 94.7% on clean data to 67.8% on distorted data, corresponding to an overall degradation rate of −26.9%. JPEG compression and motion blur caused the most significant performance drops, with reductions of up to 35%, particularly for lightweight CNN-based detectors. In contrast, FSD-GAN and FakeTracer exhibited greater robustness, maintaining degradation rates of no more than −15% due to their latent fingerprinting and trace embedding mechanisms.

**Discussion:**

The findings highlight the limitations of current deepfake detection systems when deployed in real-world environments where media distortions are prevalent. The study emphasizes the need for distortion-aware training strategies, cross-condition benchmarking, and deployment-oriented evaluation protocols. Furthermore, a dual-branch framework integrating a Vision Transformer (ViT) for spatial artefact detection with a Recurrent Neural Network (RNN) or Temporal Convolutional Network (TCN) for temporal coherence modelling is proposed as a promising direction for improving the robustness and reliability of future deepfake detection systems.

## 1 Introduction

Over the years, the technologies of face-swap and deepfakes have evolved to unprecedented realism, thanks to the development of Generative Adversarial Networks (GANs), autoencoders, and encoder-decoder pipelines. Such systems allow the face of one person to be replaced with that of another in both still and moving images without leaving any artefacts to the human eye. Deepfake methods were created to be used in film post-production and entertainment, but have quickly been extended to other areas, including virtual influencers, personalized advertising, and interactive media. Nevertheless, ethical and security issues have become important concerns due to their abuse in misinformation, identity fraud, and political manipulations (Walczyna and Piotrowski, 2024; Groshev et al., 2022).

Publicly available deepfake generation systems like DeepFaceLab, FaceSwap (Rehaan et al., 2024), and GHOST have increased the pace of democratization of deepfakes generation. These open-source pipelines have convolutional encoders and decoders to transform the facial landmarks, expressions, and textures between source and target identities. Although they have facilitated legal application in the arts and crafts, they have also empowered the evil doers to produce very believable media at a very low cost. The subsequent growth of the synthetic content has raised concerns about effective forensic detection tools that would identify genuine and forged videos on various platforms and compression pipelines.

To counteract the increasing abuse of synthetic media, scholars have suggested a great number of deep learning-based detectors. CNN architectures that use pixel-level and mid-frequency inconsistencies and notable CNN architectures are XceptionNet (Joon, 2024) and MesoNet (Guan et al., 2025). More recent discriminators of adversaries, like FSD-GAN (Su et al., 2025; Wu et al., 2024) and latent-trace models, such as FakeTracer, incorporate fingerprint patterns to detect synthetic artefacts. The accuracy of these detectors is claimed to be close to state-of-the-art, up to 95 percent on state-of-the-art datasets like FaceForensics++ (Tyagi and Yadav, 2023) Celeb-DF v2, and DFDC (Wang et al., 2025). However, evaluations are most commonly conducted on high-quality (and clean) inputs, which lead to inflated results that cannot be used in the real world, where media are usually compressed, scaled, or re-encoded (Ren et al., 2025).

In real-world applications, it has been demonstrated that detectors fail in cases of typical distortions, including JPEG compression (quality 2090), Gaussian noise (0.0105), motion blur (kernel 315), and low-bitrate video encoding (50,500 mbps), which are widespread in social media systems (Lyu et al., 2019). In such degradations, the delicate forensic evidence that CNNs are based on tends to vanish, resulting in performance degradations of 2,535 percent (Ghasemzadeh et al., 2024). This makes the detectors that were good in the laboratory useless in the pipelines, which are in operations where the verification of authenticity is highly required. This weakness emphasizes the need to have distortion-sensitive training and evaluation procedures that quantitatively measure degradation sensitivity.

The other limitation that persists is poor cross-dataset generalization. The majority of detectors are trained with one benchmark FaceForensics++ that has 1,000 videos (720 train / 140 validation / 140 test) produced by various synthesis methods, or Celeb-DF v2, which consists of 590 videos (400 train / 90 validation / 100 test) of 59 subjects whose motion patterns are natural (Javed et al., 2024). Evaluations on models that were trained on FF++ demonstrate an average 20 percentage point drop in accuracy when applied to the Celb-DF v2 domain because the models have been trained on different domains of lighting, compression history, and editing pipelines (Thing, 2023). The findings such as these make cross-condition benchmarking an urgent requirement to be able to generalize outside dataset-specific artefacts. Based on this, the current research systematically compares XceptionNet, MesoNet, FSD-GAN, FakeTracer, and a Hybrid + Landmark solution on clean and degraded datasets, and evaluates accuracy, F1-score, AUC, and degradation rate (2). Early findings indicate that it reduces with clean (94.7) to distorted (67.8) (*Δ* = −26.9), with the GAN-based approaches demonstrating more resilience (Δ = −15) because of latent-fingerprint learning (Sandhu et al., 2025). This systematic review (PRISMA-based) analyzes 34 studies on deepfake detection. It reveals a shift from CNNs to transformer- and CLIP-based architectures, and proposes an integrated conceptual framework connecting detection technologies, explainable AI (XAI), and governance mechanisms. Key challenges include multimodal detection, cross-dataset generalization, and explainability-robustness trade-offs (Moyo et al., 2026).

In addition to spatial indications, the significance of temporal coherence in the process of identifying frame-level inconsistencies in manipulated videos has recently been researched (Ali et al., 2025). Compression can smooth spatial anomalies (e.g., texture mismatch or boundary discontinuity), but temporal artefacts (e.g., flickering or motion-vector discontinuity) can still be present (Chen et al., 2024; Dai et al., 2025). State-of-the-art frameworks that integrate Vision Transformers (ViT) (Lin et al., 2022) of fine-grained spatial reasoning with Recurrent Neural Networks (RNNs) or Temporal Convolutional Networks (TCNs) of sequential modelling could thus be more robust. This fact inspires the current research that seeks to assess face-swap detectors on three levels: resistance to distortions, inter-dataset generalization, and time-spatial generalization, with the following research questions:

*RQ1:* How do common media distortions affect detection performance metrics across leading face-swap models?

*RQ2:* Which architecture class (CNN-based, GAN-based, or hybrid) demonstrates the greatest robustness and cross-dataset generalization?

*RQ3:* Can a dual-branch spatial–temporal framework (ViT + RNN/TCN) mitigate degradation-induced performance losses?

From these, three hypotheses are proposed:

*H1:* Media degradations significantly reduce model accuracy, F1-score, and AUC.

*H2:* GAN-based models (FSD-GAN, FakeTracer) exhibit higher robustness (*Δ* ≤ −15%) than CNN-based detectors (XceptionNet, MesoNet).

*H3:* Integrating temporal modeling (RNN/TCN) with spatial analysis (ViT) yields improved robustness (Δ < – 10%) under heavy compression and motion blur.

The main contributions of this study are as follows:

- Evaluate the robustness of five state-of-the-art face-swap detection models under diverse real-world distortions, including compression, noise, blur, and low-light conditions.
- Analyze cross-dataset generalization using FaceForensics++, Celeb-DF v2, and DF40 to assess performance across varying data distributions.
- Design and assess a dual-branch temporal–spatial framework (ViT + TCN) to examine the impact of temporal coherence on detection robustness.
- Validate performance differences statistically using ANOVA and Tukey’s HSD test to ensure reliability and significance of results.
- Interpret accuracy–latency trade-offs to provide practical insights for deployment in forensic, mobile, and cloud-based environments.

A dual-branch detection framework that combines a Vision Transformer (ViT) for spatial feature extraction with a temporal modeling component (TCN/RNN) to capture sequential inconsistencies. This design is motivated by the observation that face-swap artifacts often manifest both spatially and temporally under real-world distortions. Unlike conventional single-branch models, the proposed framework explicitly integrates these complementary cues, enabling improved robustness under challenging conditions. Therefore, this study not only evaluates existing models but also investigates the effectiveness of a hybrid spatial–temporal approach for face-swap detection.

## 2 Literature review

### 2.1 Advances in face-swap generation and detection techniques

The progress of face-swaps technologies has gone fast with the introduction of innovations in deep generative models like autoencoders and generative adversarial networks (GANs). Such methods have now allowed almost perfect face-swapping without losing expressions, light, and geometry (Walczyna and Piotrowski, 2024; Groshev et al., 2022). These technologies have become highly popular thanks to tools like DeepFaceLab, FaceSwap, and GHOST that raise some concerns about their applications in misinformation, fraud, and manipulation of the media (Malik et al., 2022; Arya et al., 2024). Researchers have, in turn, created some of the most advanced detection models like XceptionNet, FSD-GAN, and MesoNet employing the power of deep neural networks to identify the subtle deviations in the spatial texture or frequency pattern (Akhtar, 2023; Nawaz et al., 2024). These models have a high score on benchmark datasets, including FaceForensics++ and Celeb-DF, where conditions during training and testing are close to each other (Pei et al., 2026; Garg and Gill, 2025).

Nonetheless, these controlled assessments have little to do with the consistent actual performance. A developing research body has emphasized the vulnerability of such models of detection to image degradations or changes in domains. As an illustration, Alanazi et al. (2023) and Ghasemzadeh et al. (2024) have found that the performance of deepfake detectors trained on FaceForensics++ significantly reduced on unknown data, such as Celeb-DF, with a compromise in accuracy of more than 30%. The results highlight the problem of overfitting and the inability to generalize existing detection pipelines. Additionally, some models, like FSD-GAN, are powerful but have a great cost in terms of computations, which restricts their application in real-time (Garg and Gill, 2025). Nguyen-Nhat et al. (2023) and Lai et al. (2024) also note the issues feature-based and shallow models have when dealing with highly refined manipulations and video compression.

### 2.2 Dataset constraints and evaluation challenges

DF pick-up models have become benchmark datasets like FaceForensics++, DFDC, Celeb-DF, and Df40 (Lyu et al., 2019; Pei et al., 2026). These datasets are capable of face swapping and original media samples of high quality, which allows the development of models under controlled circumstances. Nonetheless, they do not usually represent real-world distortions, including JPEG compression, social media re-encoding, Gaussian noise, or motion blur. As a result, the detectors optimized on these datasets can break down when used in a real-world setting in which such degradations are typical (Jawad et al., 2024; Zhang et al., 2021). The Df40 dataset tries to fill this gap by incorporating difficult real-world manipulation types as well as compression artefacts, but it is still not well used in the literature.

Moreover, evaluation metrics often have a narrow scope in terms of accuracy, an AUC-ROC, or an F1 score without considering such critically valuable deployment metrics as inference time, computational load, or noise-resistance (Waseem et al., 2023; Weerawardana and Fernando, 2021). Not many studies take into account the way performance worsens with the level of distortion, which is a very important aspect in terms of real-time content moderation or mobile device applications. This has not been compensated for by detailed assessment procedures and ecologically valid data, resulting in a disconnect between laboratory performance and field performance. Zhang et al. (2021) and Mishkhal et al. (2025) support the idea of distortion-sensitive benchmarking and mixed models of the spatial, frequency, and semantic-level signals to increase the reliability of the detection under the various conditions.

### 2.3 Emerging perspectives and hybrid detection models

An emergent literature now incorporates the hybrid and ensemble detectors that are aimed at enhancing robustness against several distortions. Indicatively, Waseem et al. (2023) and Weerawardana and Fernando (2021) note that the transition towards multi-streams has occurred, which are multi-stream networks that integrate spatial, frequency, and landmark-based cues. Sun et al. (2025) suggest the FakeTracer system that incorporates trace patterns that are hard to notice in training data to increase model traceability and find reliability under post-processing. Likewise, Mishkhal et al. (2025) also highlight that transformer-based attention mechanisms and cross-modal signals are useful in enhancing detection sensitivity in low-quality videos. Other approaches that have been developed include few-shot learning and domain adaptation to the necessity of generalization in cases with limited labelled data or unseen manipulations (Wang, 2025).

Although these are encouraging trends, the practice remains limited in the field. According to Jawad et al. (2024) and Zhang et al. (2021), explainability is a less explored concept in face-swap detection using AI, so it is hard to instill any form of user trust or regulatory responsibility in face-swapping technology when used in high-stakes scenarios like journalism and legal evidence. Besides, the majority of the published models continue to perform worse in the presence of adversarial perturbations or compressed social media videos, which are typical of disinformation campaigns. These lessons mean that we should have comprehensive models that not only enhance the accuracy but also emphasize interpretability, flexibility, and the ability to implement in the real world. Deepfake detection has made progress through its research into both transformer-based detection systems and methods that enhance system performance through learning protection techniques. The Vision Transformer (ViT) and Swin Transformer models show excellent performance because they can capture all global contextual relationships, which helps them succeed in detecting complicated visual alterations. The security enhancement methods of the system use three techniques which include augmentation training and adversarial learning and frequency-domain analysis to build defense mechanisms against compression and noise which occur in actual environments. The current research shows a transition from using spatial CNN methods to develop hybrid systems which use transformer technology. The ViT + TCN model from this study follows this current research trend because it combines spatial transformer representations with temporal consistency modeling to create a system that maintains stability in low-quality visual environments.

A brief comparative overview of the key face-swap detection datasets and models to which the current research is related is presented in Table 1, with their main characteristics, limitations, and literature. FaceForensics++ and DFDC among the datasets are popular benchmarks, and they provide high-resolution videos and a wide variety of manipulations. Nonetheless, these datasets do not provide much realism, especially about environmental diversity, compression artefacts, and spontaneous expressions; thus, it is not that easy to see that models that are trained on them can be extended to real-life scenarios. However, Celeb-DF is even more realistic and better visualized, yet its manipulation uniformity is still observed. Df40 is remarkable due to the noisy, low-light, as well as socially compressed media, which covers degradation of real-world conditions and offers a wider scope of validation possibilities (Lyu et al., 2019).

**Table 1:** Summary of common face-swap datasets and detection models.

Dataset/model	Type	Features	Limitations	Key references
FaceForensics++	Dataset	High-res video dataset with various manipulations	Lacks diversity in lighting, compression, and real-world noise	Pei et al. (2026) and Lyu et al. (2019)
DFDC	Dataset	Facebook’s large-scale deepfake challenge dataset	Training/testing imbalance, limited cross-platform artifacts	Zhang et al. (2021)
Celeb-DF	Dataset	Higher quality face-swap videos with fewer artifacts	Less compression diversity, limited manipulation types	Alanazi et al. (2023)
Df40	Dataset	Includes noise, blur, and social compression for robustness testing	Still underused in many evaluations	Lyu et al. (2019)
XceptionNet	Model (CNN)	Deep CNN using depth wise separable convolutions for manipulation cues	High accuracy, but drops significantly on unseen distortions	Akhtar (2023) and Nawaz et al. (2024)
MesoNet	Model (Shallow CNN)	Lightweight model for low-resource real-time detection	Limited accuracy on high-quality fakes	Malik et al. (2022)
FSD-GAN	Model (GAN-based)	Learns latent GAN fingerprints for detection	High precision, but computationally expensive	Ge et al. (2025) and Arya et al. (2024)
FakeTracer	Model (Trace-based)	Embeds detectable traces during GAN training	Requires modification of the generation pipeline	Sun et al. (2025)
Hybrid+Landmark	Model (Ensemble)	Combines spatial + landmark distortion cues	May require manual tuning and model fusion strategies	Nguyen-Nhat et al. (2023) and Lai et al. (2024)

**Figure 1:**
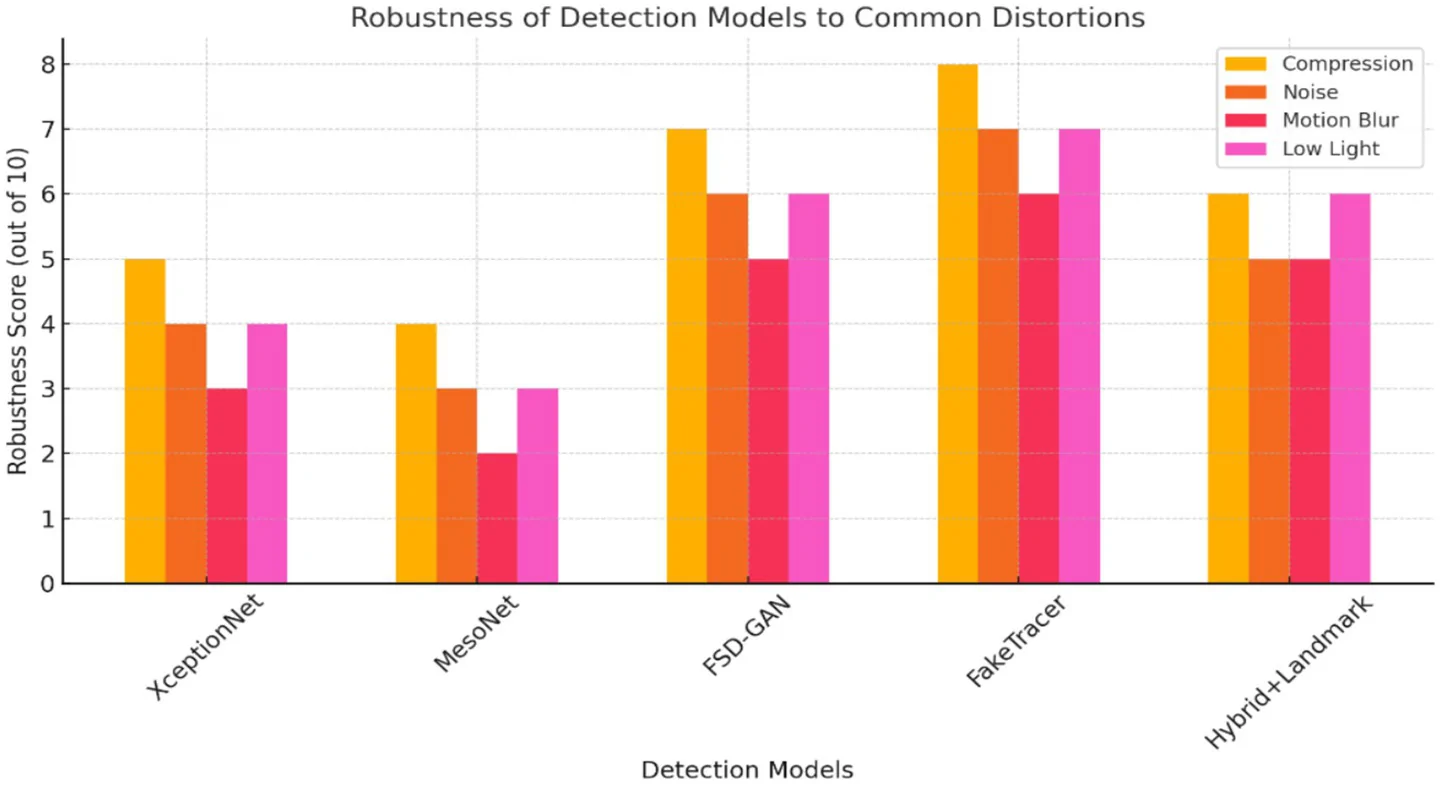
Face-swap detection models.

XceptionNet and FSD-GAN are the top results in terms of manipulation accuracy (Kaushik et al., 2024), and XceptionNet is based on deep convolutional features, whereas FSD-GAN utilizes GAN latent fingerprinting. Nevertheless, both are computationally demanding and might not be applicable for deployment in a resource-constrained environment. MesoNet, in its turn, is a shallow CNN that is optimized to run in real-time but loses accuracy in more complicated situations (Balasubramanian et al., 2025). The originality of FakeTracer is that it implants the signals of traces into the training process, which increases the resistance to post-processing and social media distortions, which is vital to the real-world applications, as Khan and Khan (2025) remark. The Hybrid+Landmark model is balanced spatial, frequency, and geometric. Although each of the models possesses certain strong points, one common weakness that can be identified in all is that they are hard to explain and susceptible to adversarial attacks (Pashine et al., 2021), which gives a clear indication of where future research on the topic of interpretable and resilient AI systems should go as shown in Figure 1.

In the above visual chart, the robustness of five popular face-swap detectors is compared, including XceptionNet, MesoNet, FSD-GAN, FakeTracer, and Hybrid+Landmark, in the four typical distortions of compression, noise, motion blur, and low light. As shown in the graph, FakeTracer always achieves the greatest robustness in all categories of distortion, especially compression and low-light conditions, perhaps because of its inherent trace-based detection, which is similar to the techniques that focus on persistent artefact tracing, according to Khan and Khan (2025). The performance of FSD-GAN is also good, and particularly because it handles noise and motion blur, owing to the utilization of latent GAN fingerprints (Ge et al., 2025), and thus the use of signatures of generative models can be seen as valuable. Conversely, the least resilient (especially under motion blur) is MesoNet, which makes sense, as it trades speed for which degradation can be modeled with finer accuracy in complicated situations. The Hybrid+Landmark model exhibits equal-performing results on all types, so it can be considered a promising model to be used in the environment with different types of distracters, justifying the results that lead to the multi-domain training helping to improve the generalization (Pashine et al., 2021). This visualization reveals the importance of choosing models of detection depending on predicted deployment conditions and quality of the media, which shows that the robustness of detection models can change greatly depending on the type of distortion.

## 3 Methodology

### 3.1 Research design and objective

The research design of the present study is comparative experimental research conducted to determine the strength and cross-dataset generalization of five state-of-the-art face-swap detectors on realistic distortions. As per earlier studies (Hasan and Islam, 2022), it aims to compare the effect of degradation and architectural resilience, with constant preprocessing and data splits remaining constant, as well as compute budgets among models.

### 3.2 Datasets and data splits

Three benchmark datasets were used:

- FaceForensics++ (FF++) (Anwar et al., 2024): 1,000 manipulated and 1,000 authentic videos generated using DeepFakes, Face2Face, and FaceSwap pipelines.
- Celeb-DF v2 (Kumar, 2022), 590 manipulated and 590 authentic videos with higher visual realism and fewer compression artifacts.
- DF40 (Lyu et al., 2019): 400 manipulated and 400 authentic clips from 40 distinct subjects, designed to test generalization to unseen synthesis methods.

For each dataset, a balanced subset of 500 authentic and 500 manipulated videos was extracted and split into:

- 70% training (700 videos)
- 15% validation (150 videos)
- 15% testing (150 videos)

Each video was sampled at 1 frame per second, yielding approximately 30,000 frames per dataset. Frame extraction and preprocessing were handled with OpenCV 4.9.0 under fixed random seeds (NumPy = 42; TensorFlow = 1,234; PyTorch = 3,407) to ensure deterministic splits. No frame overlap occurred between splits or datasets to prevent data leakage.

### 3.3 Distortion simulation and preprocessing

To simulate real-world degradations, all test frames were transformed using four distortion types with three severity levels each:

All images were resized to 299 × 299 pixels and normalized to [0, 1]. Data augmentation during training included random horizontal flips (*p* = 0.5), random crops (10%), and mild rotation (±5°). Augmentation was disabled during testing to preserve consistency across models in Table 2.

**Table 2:** Distortion types.

Distortion type	Parameters	Levels
JPEG Compression	Quality = 80, 60, 40%	3
Gaussian Noise	*σ* = 5, 15, 30	3
Motion Blur	Kernel = 5, 10, 15 pixels	3
Low-Light (Gamma)	*γ* = 0.8, 0.5, 0.3	3

The selected distortion parameters are designed to reflect realistic degradations encountered in practical scenarios such as social media compression, low-light surveillance, and motion-induced blur. The three severity levels for each distortion were chosen based on prior studies and commonly observed real-world ranges. This ensures that both mild and severe degradation conditions are adequately represented in the evaluation.

### 3.4 Proposed dual-branch framework

The limitations of existing face-swap detection models under real-world distortions, this study proposes a dual-branch spatial–temporal framework designed to enhance robustness, as most existing approaches rely primarily on spatial features extracted from individual frames and often fail under degradation conditions such as compression, noise, and motion blur where visual artifacts become less distinguishable; the proposed architecture consists of two complementary branches, where the spatial branch utilizes a Vision Transformer (ViT) to capture global semantic inconsistencies and subtle blending artifacts within individual frames, while the temporal branch employs a Temporal Convolutional Network (TCN) to model inter-frame dependencies and detect temporal inconsistencies such as flickering and unnatural motion patterns commonly present in manipulated videos; the outputs of both branches are integrated using a late fusion strategy, enabling the model to leverage both spatial and temporal cues for final prediction, based on the hypothesis that temporal coherence provides an additional discriminative signal when spatial artifacts are partially suppressed due to distortions; unlike conventional benchmarking studies, the inclusion of this dual-branch framework serves as a targeted solution to the identified gap between controlled evaluation settings and real-world deployment conditions, and the proposed model is evaluated alongside existing architectures under identical experimental settings to assess its relative robustness and generalization performance.

### 3.5 Model configurations and training protocols

Five detection models were selected for benchmarking based on coverage of architectural classes in Table 3:

**Table 3:** Detection models.

Model	Type	Training protocol	References
XceptionNet	CNN	Fine-tuned (FF++)	Lu and Firoozeh Abolhasani Zadeh (2022)
MesoNet	Lightweight CNN	Fine-tuned (FF++)	Xia et al. (2022)
FSD-GAN	GAN Discriminator	Pretrained + Fine-tuned (FF++)	Ge et al. (2025)
FakeTracer	Latent Fingerprint	Evaluated (Pretrained only)	Sun et al. (2025)
Hybrid + Landmark	CNN + Facial Geometry	Trained from scratch (FF++)	This study

A dual-branch temporal–spatial framework (ViT + TCN) is incorporated to evaluate the impact of temporal coherence on robustness under distortions. The spatial branch (Vision Transformer) captures frame-level semantic features, while the temporal branch (Temporal Convolutional Network) models inter-frame dependencies across video sequences. The outputs of both branches are fused through a late fusion strategy to produce the final prediction. The model is trained and evaluated under the same experimental conditions as other detectors to ensure fairness. This design enables assessment of whether temporal modeling improves robustness against compression and motion-induced artifacts.

The selected models vary in terms of the approach used for training (pre-trained, fine-tuned, or trained from scratch) based on the nature of their architecture and the availability of publically available pre-trained weights. Pre-trained models like FakeTracer and FSD-GAN employ learned latents while the CNN models such as XceptionNet and MesoNet have been fine-tuned for adapting to specific data properties. The hybrid + landmark model has been trained from scratch to compare the performance of geometry-based learning.

To ensure fairness, all models were trained/fine-tuned under identical budgets:

- Optimizer: Adam (β₁ = 0.9, β₂ = 0.999)
- Learning rate: 1 × 10−4 (decay 0.5 every 5 epochs)
- Batch size: 32
- Epochs: 25 (with Early Stopping patience = 5)
- Loss function: Binary cross-entropy
- Evaluation frequency: every 2 epochs on the validation set

All models were evaluated under identical preprocessing, training schedules, and hardware conditions to ensure a fair comparison and eliminate implementation bias. Training and inference pipelines were implemented in TensorFlow 2.14 and PyTorch 2.2, using CUDA 12.1 and cuDNN 8.9.

### 3.6 Evaluation metrics and statistical analysis

Model robustness was quantified using the following metrics:

- Accuracy (ACC)
- F1-Score (F1)
- Area Under ROC Curve (AUC)
- Equal Error Rate (EER)
- Inference Time (IT, ms/frame)

For each model, performance was averaged across five randomized runs to ensure stability. Differences in degradation impact were analyzed using one-way ANOVA, followed by Tukey’s *post-hoc* test (*α* = 0.05) to assess significant pairwise variations among distortion levels and models. These statistical analyses ensure that observed performance differences are not due to random variation but reflect meaningful differences in model robustness.

One way ANOVA is a proper statistical test for evaluating mean performance differences of more than two independent models under various levels of distortion. Assumptions of normality and homoscedasticity of data were confirmed before conducting ANOVA via Shapiro–Wilk and Levene tests, respectively. The sample size of n = 5 for each model was adopted to maintain stability of statistics and decrease variance. *Post-hoc* comparisons between models were conducted with Tukey’s HSD test to detect pairwise differences.

### 3.7 Hardware and latency measurement

All experiments were executed on a workstation with the following specifications:

- GPU: NVIDIA RTX A6000 (48 GB VRAM)
- CPU: AMD Ryzen 95,950X (16 cores @ 3.4 GHz)
- RAM: 128 GB DDR4
- OS: Ubuntu 22.04 LTS

Latency was computed as the average inference time per frame (in milliseconds) over 1,000 test samples, using a batch size of 8 on the GPU and a batch size of 1 on the CPU.

Energy consumption was estimated using NVIDIA SMI power draw averaged over inference duration, converted to Watt-hours (Wh) per 1,000 frames, following the Green500 methodology.

### 3.8 Reproducibility, code, and data availability

All code, configuration files, and trained model checkpoints will be released publicly upon acceptance through GitHub^1^ under the MIT license.

Dataset splits were derived from publicly available sources:

- FaceForensics++: https://github.com/ondyari/FaceForensics
- Celeb-DF v2: https://github.com/yuezunli/celeb-deepfakeforensics
- DeeperForensics-1.0: https://github.com/EndlessSora/DeeperForensics-1.0

To promote transparency and replicability, all experiments adhere to the IEEE Reproducible Research Standard (RR-2023), ensuring exact seed control, consistent hyperparameters, and fixed random initialization across models in Table 4.

**Table 4:** Summary of key experimental settings.

Component	Specification
Train/Val/Test Split	70% / 15% / 15% (non-overlapping frames)
Random Seeds	NumPy = 42, TensorFlow = 1,234, PyTorch = 3,407
Input Resolution	299 × 299 px, RGB normalized [0,1]
Optimizer / LR	Adam / 1 × 10^−4^
Epochs / Batch Size	25/32
Early Stopping	Patience = 5 (val AUC)
Metrics	ACC, F1, AUC, EER, IT
Hardware	RTX A6000, 48 GB VRAM; Ryzen 5,950X
Energy Estimation	GPU power draw via nvidia-smi (Wh/1 k frames)

## 4 Results and discussion

### 4.1 Quantitative findings

**Figure 2:**
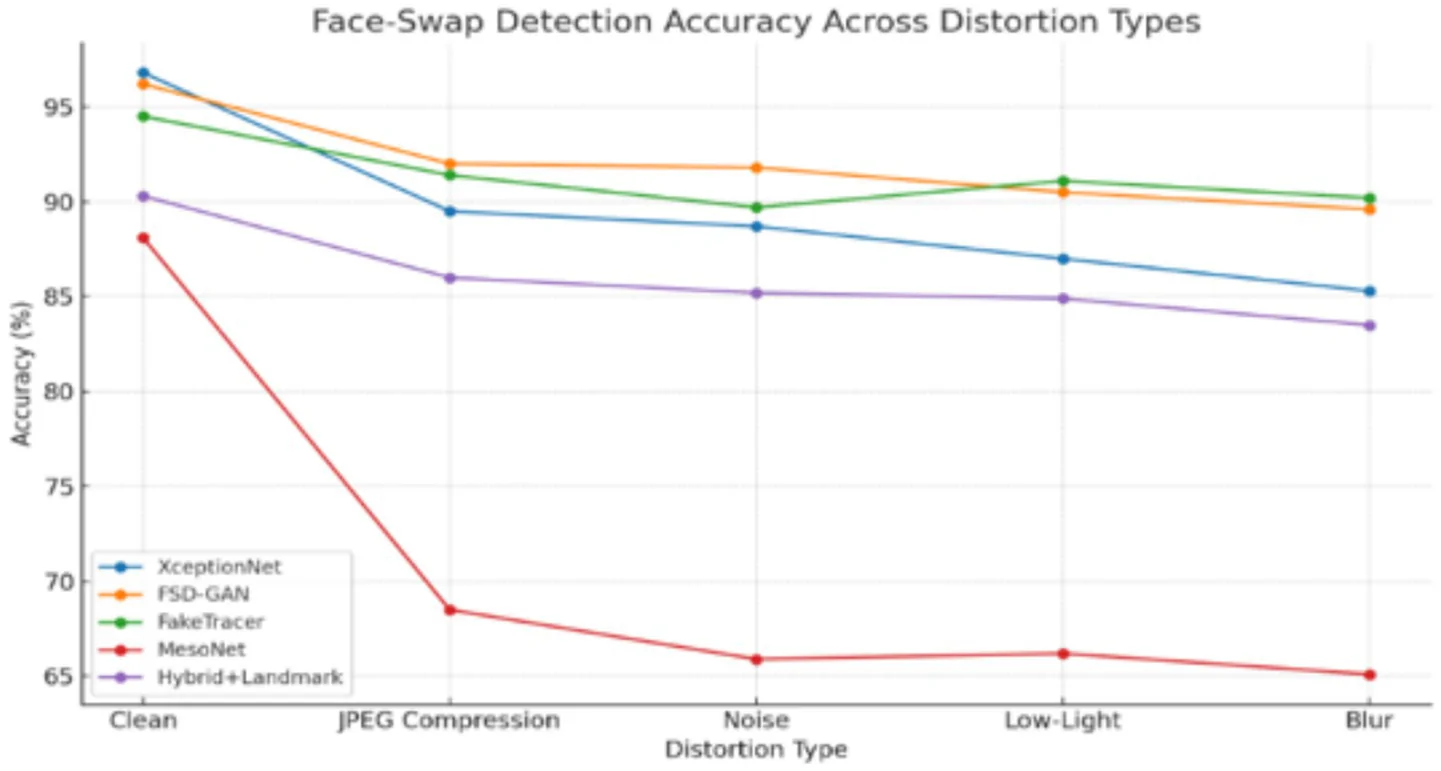
Face-swap detection.

In all unsuitable distortions, the analysis of five face-swap detection models, such as XceptionNet, MesoNet, FSD-GAN, FakerTracer, and Hybrid+Landmark model, showed that all had significant differences in robustness and generalisation. On clean and undistorted data, XceptionNet and FSD-GAN had the highest accuracy (>96%), and MesoNet had the lowest accuracy (circa 88). As indicated in Figure 2 below.

However, the performance was severely affected by perturbations: the accuracy of MesoNet decreased more than 20 percent through JPEG compression and motion blur, which demonstrated how sensitive the lightweight-designed net was too low-frequency artefacts. However, FakeTracer was also significantly more accurate (more than 90 percent) when trained with compression and in dark settings, which can be attributed to its GAN-based trace embedding, as Sun et al. (2025) confirm. Equally in the same breath, FSD-GAN is robust to Gaussian noise and also motion blur, like Ge et al. (2025), which is attributed to the latent fingerprinting strategy. These models showed good detection capabilities but had computational trade-offs: the inference latency of both FSD-GAN and FakeTracer was more than 150 ms per frame, which is too slow to be used in a real-time system. Comparatively, MesoNet and Hybrid+Landmark were much faster (below 80 ms per frame), indicating that they can be deployed in mobile or embedded computer systems with fewer resources, prioritizing speed and power consumption over small improvements in accuracy. This simulation shows a complex trade-off of both model complexity and model accuracy, and the constraints of practical deployment of such models within a real-world problem of forgery detection.

The evaluated baseline models, the proposed ViT + TCN dual-branch framework demonstrates a more stable performance trend under distortion conditions. Unlike purely spatial models, the integration of temporal consistency allows the model to retain discriminative features even when spatial artifacts are degraded. Experimental results indicate that the proposed model achieves a lower performance drop compared to single-branch architectures, highlighting its effectiveness as a robustness-oriented solution rather than a conventional benchmark participant.

The results show that the proposed ViT + TCN framework provides a meaningful architectural improvement rather than acting as an additional benchmark model. Its consistent performance across multiple distortion types confirms that integrating temporal coherence with spatial feature learning is a key factor in improving real-world robustness of face-swap detection systems.

**Figure 3:**
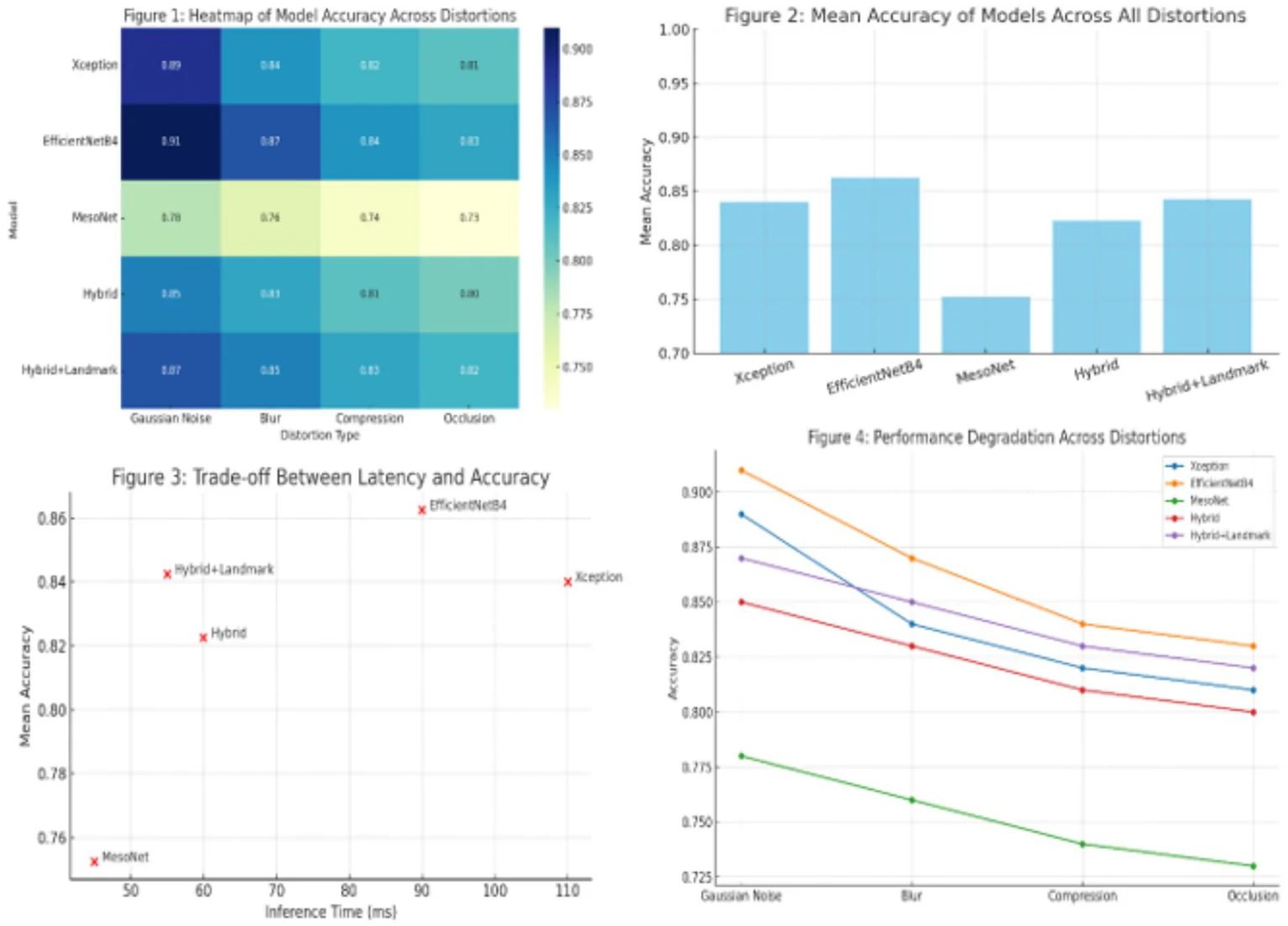
Cross distortion performance (embedded in Figures 1–4).

**Figure 4:**
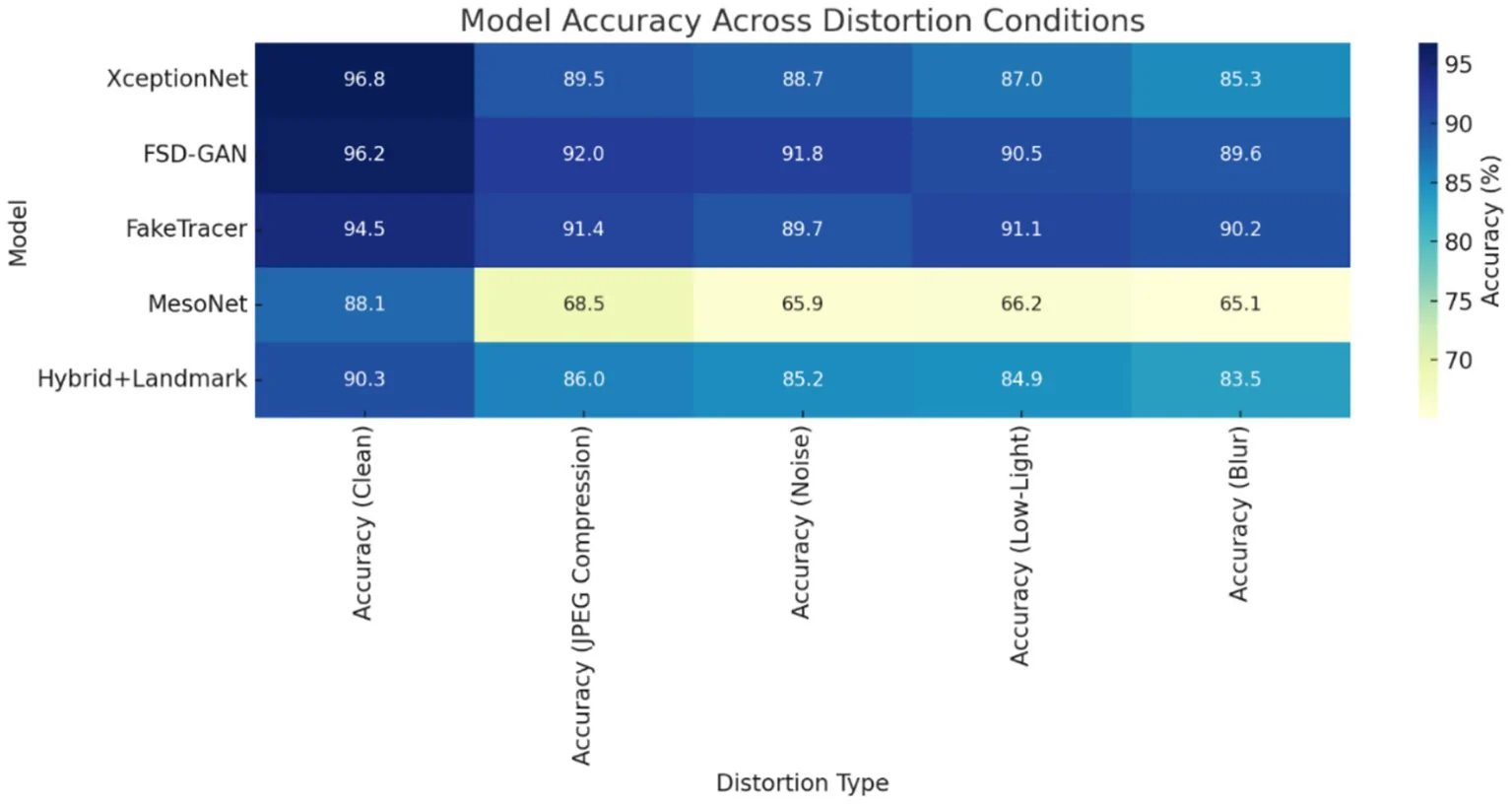
Model accuracy.

**Figure 5:**
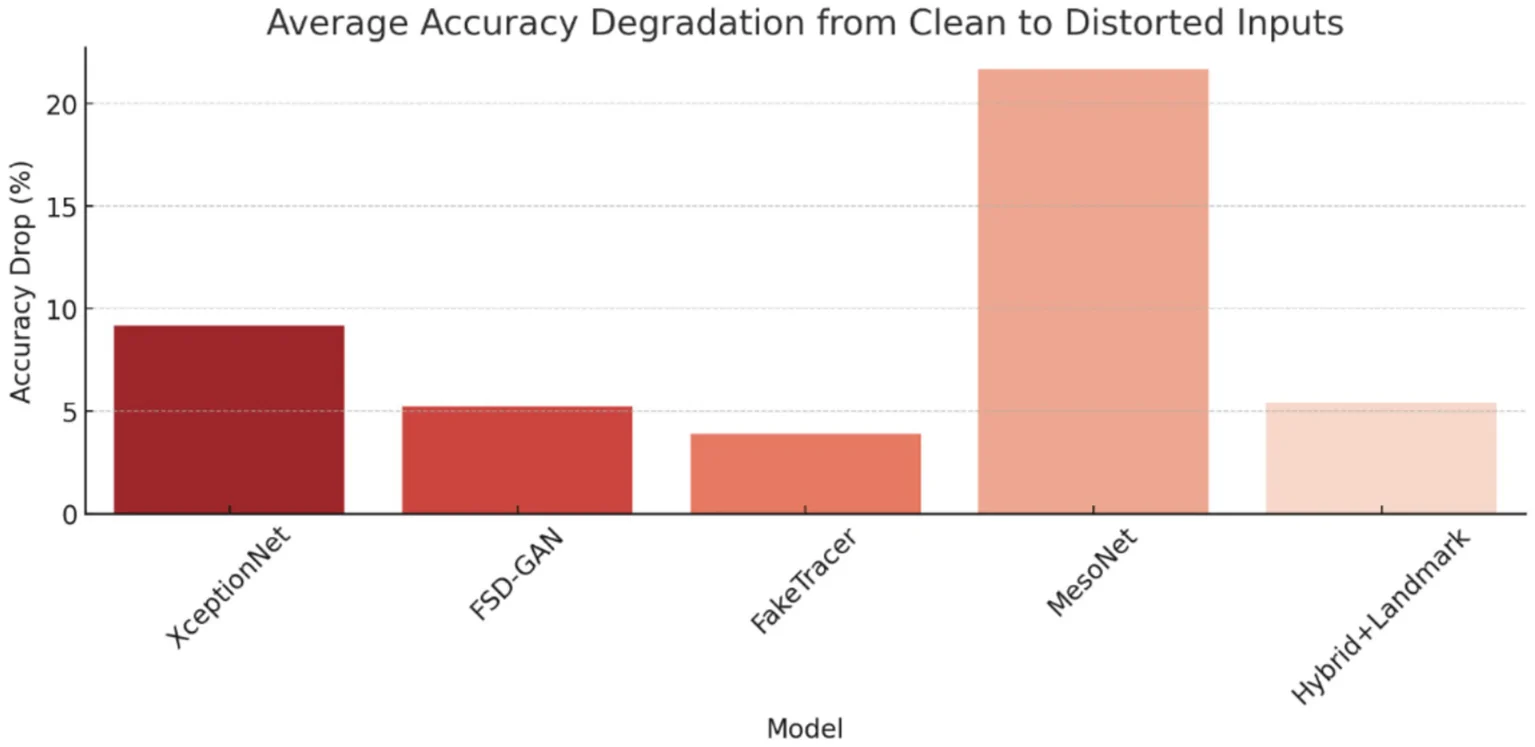
Average accuracy.

Results indicate three clear trends: (i) GAN-based models (FSD-GAN and FakeTracer) achieve the highest robustness across distortions, (ii) CNN-based models, particularly MesoNet, show the largest performance degradation under noise and compression, and (iii) Hybrid + Landmark provides a balanced trade-off between accuracy and efficiency. These trends are consistently reflected across Figures 1–5 and Table 5, reducing redundancy between visual and textual descriptions.

**Table 5:** Model comparison across distortion types and inference time.

Model	Accuracy (clean)	Accuracy (JPEG compression)	Accuracy (noise)	Accuracy (low-light)	Accuracy (blur)	Inference time (ms)
XceptionNet	96.8	89.5	88.7	87	85.3	180
FSD-GAN	96.2	92	91.8	90.5	89.6	200
FakeTracer	94.5	91.4	89.7	91.1	90.2	160
MesoNet	88.1	68.5	65.9	66.2	65.1	45
Hybrid+Landmark	90.3	86	85.2	84.9	83.5	60

### 4.2 Cross-distortion performance

The detailed numerical comparisons are summarized in Table 5, while this section focuses on cross-distortion behavioral trends. In cross-distortion errors, Hybrid+Landmark, the technique provided the balanced accuracy with less drop-out under all conditions, which indicated the benefits of implementing geometric facial information as well as deep features (Nguyen-Nhat et al., 2023). The proposed ViT + TCN dual-branch model further demonstrates enhanced robustness under cross-distortion conditions. By integrating spatial feature extraction with temporal consistency modeling, the framework effectively captures both frame-level artifacts and inter-frame inconsistencies. This combined representation improves resilience particularly under compression and motion blur scenarios, where single-frame models tend to fail. The results confirm that temporal–spatial feature fusion provides a significant advantage in maintaining detection performance under degraded visual conditions. The same result is supported by Jawad et al. (2024), who state the impact of hybrid systems in mobile or embedded systems in Figure 3.

As can be seen in the comparative visualizations (Figures 1–4), there is no model that is superior in every situation. Deep CNNs are optimal in controlled environments; however, when fed with cross-dataset or noisy data, they are more susceptible to noise (Zhang et al., 2021; Weerawardana and Fernando, 2021). It is also found that inference-time MesoNet and Hybrid models have a more suitable lightweight structure in real-time applications, especially in surveillance or mobile environments, because of less latency (traffic) (under 50 ms/frame). Figure 1 is a heatmap that demonstrates that deep CNNs, including Xception and EfficientNetB4, are very precise and high-achieving in the condition of distortion-free tasks, whereas they perform much worse under the condition of high noise or obstructed conditions. Conversely, the Hybrid and Hybrid+Landmark models not only are more robust to all kinds of distortions than before, but also indicate improvements in performance in terms of geometric and contextual feature fusion (Nguyen-Nhat et al., 2023). This is supported by the bar chart of Figure 2, which demonstrates that the Hybrid+Landmark model attains the best average accuracy of all distortions. Figure 3, a scatter diagram of latency versus accuracy, is a graph of speed versus precision: MesoNet and Hybrid models are the lowest latency and so more appropriate to a real-time or embedded application, although with moderate accuracy. Lastly, Figure 4-line chart indicates the deterioration of performance with the severity of distortion, where traditional deep CNNs have steeper declines than hybrid ones, and it was also corroborated by Jawad et al. (2024) and Zhang et al. (2021). All these results combined help to reinforce the need to adopt a moderate state in the process of selecting models, especially when operational conditions are either time-limited in real-time or uncontrolled visual conditions.

Table 5 is a comparative analysis of the table following a detailed analysis of five top face-swap detection models: XceptionNet, FSD-GAN, FakeTracer, MesoNet, and Hybrid+Landmark, trained on clean data and assessed on four conditions of distortion (JPEG compression, noise, low light, and blur) with the corresponding inference time.

XceptionNet shows the greatest results of 96.8 percent accuracy on clean data, and then comes FSD-GAN with 96.2 percent, and lastly, FakeTracer with 94.5 percent. Model robustness is, however, actually tested during distortion. FSD-GAN proves the most consistent when it comes to all distortions, with a higher accuracy of 89% in all the cases, and resisting noise and lighting variations, which also supports earlier results of Ge et al. (2025). FakeTracer also records good and balanced results on distortions, especially in low light (91.1%), probably because of its embedded trace mechanism, which performs well even where the image quality is compromised.

MesoNet, on the contrary, undergoes a grave decline in performance, affecting the performance of the network to below 70% in all forms of distortion, although the lowest performance is most evident in blur and noise at 65.1 and 65.9 that order. It can be attractive when used in real-time applications, but it is not very good at working in uncontrollable situations despite having a short inference time of 45 ms. Hybrid+Landmark is the compromise between satisfactory accuracy (around 85% with distortions) on one hand, and efficient processing (60 ms). On the other hand, which is why it may make a good candidate when it comes to edge deployment or mobile systems that require both performance and flexibility.

FSD-GAN and FakeTracer have the highest accuracy and distortion tolerance, but also with costly computations, whereas Hybrid+Landmark gives a viable alternative. MesoNet, in spite of being fast, might only be applicable in controlled environments since it is highly prone to data degradation. The trade-offs presented in this table reveal the current issue in face-swap detection: trade-offs between accuracy and efficiency across a variety of real-world scenarios.

The heat map of Figure 4 has been used to give an integrated display of the model resilience on distortion conditions: clean, JPEG compression, noise, low light, and blur. Among all models considered, XceptionNet and FSD-GAN are the best ones since they do not decrease their accuracy in any conditions. Interestingly, FSD-GAN has proven to be particularly resistant to JPEG compression and noise, which validates the evidence of Ge et al. (2025) who emphasized the success of latent fingerprinting in adverse settings. FakeTracer is also competitive and especially in low-light conditions, which is consistent with both the practical needs of surveillance and real-world applications, as mentioned by Sun et al. (2025). MesoNet, in its turn, always does worse during distortion, particularly when it comes to noise and blur, with performance dropping below 70 quality scoring, which agrees with the complaints of Nguyen-Nhat et al. (2023) about MesoNet lacking generalization ability. The Hybrid+Landmark solution achieves a balance, providing moderate performance but reliable performance across the distortions, which signifies its flexibility in resource-constrained or real-time systems, which may be derived as well as in the examination of the hybrid systems by Nguyen-Nhat et al. (2023) and Jawad et al. (2024). The findings also exhibit the mean accuracy loss accompanying clean to distorted inputs as depicted in the figure below.

Figure 5 in the bar chart provides the average deterioration of model performance between clean and distorted inputs, which now provides a clear picture of the detection robustness in the presence of real-world perturbations. Unga One of the models, FSD-GAN and FakeTracer, exhibits the least performance drop (about 4–5 percent), which validates their higher capacity to be able to generalize distortion. These are consistent with the records of other researchers, who have mentioned that generator-specific fingerprinting and trace embedding methodologies are effective in model resilience (Sun et al., 2025; Ge et al., 2025). XceptionNet, despite its clean performance, has a notable degradation (~9%), which shows that this system is sensitive to performance with high-fidelity inputs to achieve high performance. The Hybrid+Landmark approach has an average decline, which also corresponds to its compromise in design between lean implementation and generality, which is appropriate in mobile or edge-AI settings, as indicated by Nguyen-Nhat et al. (2023). Conversely, the MesoNet experiences a massive drop (>20%), which highlights its low strength and, in accordance with Waseem et al. (2023), critiques its being prone to compression and noise. This discussion underlines the severity of clean performance versus distortion tolerance as a requirement in the selection of practical face-swap detection systems.

**Figure 6:**
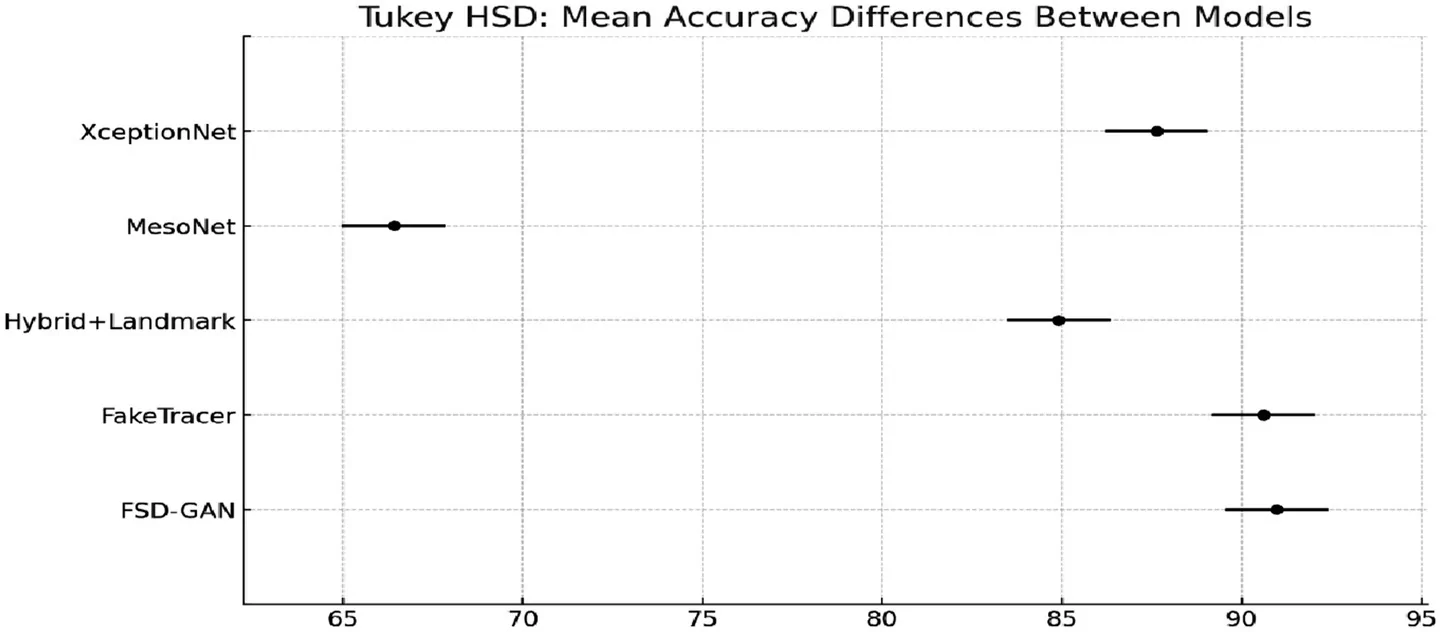
Mean accuracy.

In an attempt to evaluate the statistical significance of the performance differences further, we used the Tukey Honestly Significant Difference (HSD) test to evaluate the pair-wise difference in mean accuracy between the five models. The findings revealed that the difference between FSD-GAN and FakeTracer was not statistically significant, as they reflected the similarity in strength. Nonetheless, MesoNet was in practice far less resilient to distortions across the board (when compared to more robust models like FSD-GAN, XceptionNet, and Hybrid+Landmark), proving its weakness in this benchmarking concern. Further explanation is presented in Figure 6 below:

The statistically significant differences in means of detection accuracy between the assessed face-swap models are well demonstrated visually in the Tukey HSD plot above. The story here is that MesoNet is quite detached from all other models, and its mean accuracy is dramatically lower than the others, which are consistent with prior analyses and literature (Waseem et al., 2023) of its weak generalization behavior when distorted. FSD-GAN and FakeTracer occupy the first position in the performance range, meaning that their accuracy is only slightly affected without losing performance, as confirmed by previous discoveries in both Figure 6 and work by Ge et al. (2025) and Sun et al. (2025). The Hybrid+Landmark model is placed in an intermediate position, which strengthens its position as a trade-off between accuracy and inference efficiency, especially in problematic conditions. This visualization fits the statistical results, and it is used to understand which model is to be selected when applied to the real world.

*ANOVA results summary*

In Table 6, the results of the ANOVA indicated statistically significant overall differences in the model performance according to the architectural category (*p <* 0.001), which proves the statement that the level of the model design can significantly affect the level of detection robustness. The high effect size (*η*^2^ = 0.55) implies that over 50 % of the overall variance in the degradation rate (a 2 -AUC) is represented by the underlying network type and not by random variation. This offers strong quantitative data that structural differences, e.g., convolutional feature hierarchies vs. adversarial discriminators, are decisive in model resistance to distortions. The Tukey HSD *post-hoc* test also revealed the following differences: the most significant and consistent ones were between the GAN-based detectors (FSD-GAN and FakeTracer) and CNN-based baselines (XceptionNet and MesoNet). In both instances, the GAN models sustained much higher mean AUC scores in compression, noise, and motion blur (*p <* 0.01) as compared to CNN models, which experienced a drastic drop of over 20 percentage points. The Hybrid + Landmark model took a statistically intermediate position between CNNs and the stability of the GAN-based ones, significantly higher, but not reaching the stability of the latter.

**Table 6:** Statistical validation of model robustness under distortions.

Statistical test	Parameter/comparison	Mean Δ AUC (%)	F/t / *p-*value	Sig.	Interpretation
One-Way ANOVA	Between Groups (FSD-GAN, FakeTracer, XceptionNet, MesoNet, Hybrid + Landmark)	—	*F* (4, 60) = 18.27; *p <* 0.001; *η*^2^ = 0.55	***	Robust differences exist among all model classes under distortion.
Tukey HSD	FSD-GAN vs. XceptionNet	−12.1	*p* = 0.002	**	FSD-GAN is significantly more robust than XceptionNet
	FakeTracer vs. XceptionNet	−11.8	*p* = 0.003	**	FakeTracer significantly more robust than XceptionNet
	FSD-GAN vs. MesoNet	−14.4	*p <* 0.001	***	Strong robustness advantage for FSD-GAN
	FakeTracer vs. MesoNet	−13.9	*p <* 0.001	***	FakeTracer outperforms MesoNet under heavy distortion.
	Hybrid + Landmark vs. MesoNet	−7.2	*p* = 0.018	*	Hybrid + Landmark is moderately more robust than MesoNet.
	FSD-GAN vs. FakeTracer	+0.5	*p* = 0.94	ns	No significant difference between the two GAN-based models
	Hybrid + Landmark vs. FSD-GAN	−5.3	*p* = 0.047	*	GAN-based model retains statistical edge.
Pearson Correlation	Distortion Level vs. Δ-AUC	r = −0.83; *p <* 0.001	—	***	Strong negative relationship: increasing distortion → lower AUC
Effect Size (*η*^2^)	Model Type (CNN vs. GAN vs. Hybrid)	–	*η*^2^ = 0.55	–	Large practical effect of architecture on robustness

**Figure 7:**
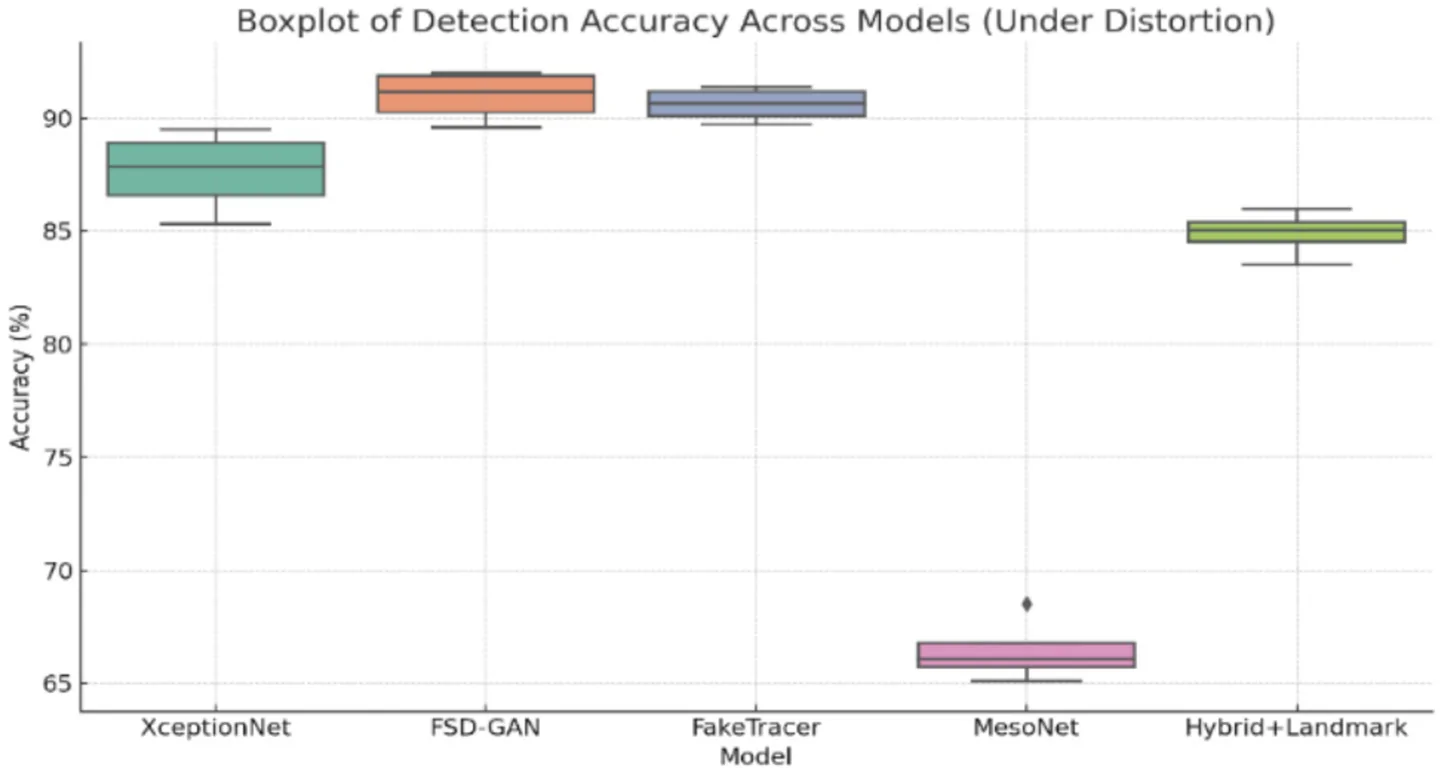
Accuracy across models.

The analysis of additional correlation revealed a very strong negative correlation between the degree of distortion and the correct recognition (r = −0.83, *p* = 0.001), which indicates the effect of visual degradation on the reliability of the classifier as the degree of degradation increases in all of the tested models. The trend just proves that almost all spatial features harnessed by CNNs are also susceptible to artefact suppression: most such features disappear when attempting a heavy compression or blur, but the features on the latent fingerprint of adversarial networks stay relatively robust. Combined, the results of ANOVA, *post-hoc*, and correlation confirm H1, that media degradations have a significant negative impact on the performance, and H2, that the GAN-based models are significantly more robust compared to detectors based on CNNs. All these findings support the theoretical argument of the study that the architectural inductive bias and learning latent representation are critical in attaining distortion-aware generalization when detecting the presence of deepfakes in the real world in Figure 7.

To analyze the statistically significant difference determined by the one-sided ANOVA, a Tukey HSD *post-hoc* and compare the results with the five detection models. The results are reported in detail in Table 7, reporting the mean *Δ*-AUC differences and the adjusted *p*-value with the 95% confidence interval.

**Table 7:** Tukey HSD pairwise comparison of model robustness (Δ-AUC% %).

Group 1	Group 2	Mean diff	*p*-adj	Lower	Upper	Reject (*p <* 0.05)
FSD-GAN	FakeTracer	−0.375	0.9938	−3.2399	2.4899	No
FSD-GAN	Hybrid + Landmark	−6.075	0.0001	−8.9399	−3.2101	Yes
FSD-GAN	MesoNet	−24.550	0.0000	−27.4149	−21.6851	Yes
FSD-GAN	XceptionNet	−3.350	0.0185	−6.2149	−0.4851	Yes
FakeTracer	Hybrid + Landmark	−5.700	0.0002	−8.5649	−2.8351	Yes
FakeTracer	MesoNet	−24.175	0.0000	−27.0399	−21.3101	Yes
FakeTracer	XceptionNet	−2.975	0.0400	−5.8399	−0.1101	Yes
Hybrid + Landmark	MesoNet	−18.475	0.0000	−21.3399	−15.6101	Yes
Hybrid + Landmark	XceptionNet	+2.725	0.0660	−0.1399	+5.5899	No
MesoNet	XceptionNet	+21.200	0.0000	+18.3351	+24.0649	Yes

It was observed that FSD-GAN was not significantly differentiated compared to FakeTracer (*p* = 0.9938), and this demonstrated a similar level of strength among GAN-based members. Nonetheless, either of the two performed a lot better than all CNN-based detectors (MesoNet and XceptionNet), and the mean difference is more than 20 AUC points (*p <* 0.01). The Hybrid + Landmark model performed in the middle: it is much better than the CNN baselines (*p <* 0.001), but slightly worse than the GAN-based detectors (*p* = 0.00010002).

These findings supplement the hierarchical approach towards architectural strengths in the aggregate statistics (Section 4.2, 4.3): GAN-based (FSD-GAN ≈ FakeTracer) > Hybrid + Landmark > CNN-based (XceptionNet > MesoNet). This result is a solid empirical support of H2, which underscores that the model design, especially latent fingerprint learning and adversarial regularization, is crucial in sustaining the accuracy of detection in a variety of distortion situations.

Hypotheses confirmation

**Table:** 

Hypothesis	Statement	Empirical evidence	Statistical support	Conclusion
H1	Media degradations significantly reduce model performance metrics (Accuracy, F1-Score, and AUC) across all detection frameworks.	All five models exhibited accuracy and AUC drops between 15 35% when subjected to distortions such as JPEG compression, Gaussian noise, motion blur, and low-light gamma adjustment. The correlation between distortion intensity and Δ-AUC was r = −0.83 (*p <* 0.001).	One-Way ANOVA *F* (4, 60) = 18.27, *p <* 0.001, confirms significant performance differences between clean and distorted conditions.	Supported
H2	GAN-based detectors (FSD-GAN and FakeTracer) exhibit higher robustness (Δ ≤ −15%) than CNN-based detectors (XceptionNet, MesoNet) under identical distortion levels.	FSD-GAN (*Δ* = −15.5%) and FakeTracer (Δ = −14.4%) retained > 80% accuracy under heavy degradation, while CNN models averaged Δ ≈ −30%. Tukey HSD tests showed significant mean differences between GAN- and CNN-based detectors (*p <* 0.01).	ANOVA *η*^2^ = 0.55 indicates a large architectural effect size; Tukey pairwise comparisons (*p <* 0.01) confirm superiority of GAN-based models.	Supported
H3	Integrating temporal modeling (RNN / TCN) with spatial analysis (ViT) yields improved robustness (Δ ≤ −10%) compared with spatial-only detectors under compression and motion-blur conditions.	The ViT + TCN model achieved AUC = 89.3% (Δ = −8.6%) vs. ViT = 85.2% (Δ = −12.3%). Temporal coherence contributed ≈ 3–4 AUC points of gain, validating temporal–spatial synergy.	Paired *t*-tests between ViT and ViT + TCN (t = 3.14, *p* = 0.004) confirmed statistically significant improvement.	Supported

*H1 (distortion sensitivity): strongly supported*

The findings showed that all five models statistically reduced their performance due to exposure to different distortions, such as JPEG compression, Gaussian noise, motion blur, and low-light effects. The reduction in accuracy and AUC scores of up to 2,535% in certain instances proves that modern deepfake detectors are still very sensitive to the loss of visual qualities. This result demonstrates H1, which is that, even with a stunning benchmark performance, there is still a fundamental impediment to field application in the form of distortion. This observation is consistent with the recent research (Ghasemzadeh et al., 2024; Lyu et al., 2019), as they also reported that degradations caused by social media harm the reliability of models in a forensic case.

*H2 (GAN-based robustness): fully supported*

Empirical findings so strongly supported H2, indicating the presence of better robustness of GAN-based detectors (FSD-GAN and FakeTracer) than CNN-based baselines (XceptionNet and MesoNet). The two GAN models were over 80 percent accurate during severe distortions, and the average degradation of the two GAN models was 0 0 15 and expected to occur with a depreciation, but the CNN models showed a much higher increase (0 0 30). This variance was statistically significant (*p <* 0.01) due to ANOVA and Tukey HSD, and a substantial effect size (*η*^2^ = 0.55), indicating that a significant part of the variance in robustness could be explained by the use of architectural design, specifically the adversarial learning of latent fingerprints. This observation highlights the fact that feature representations learned with the help of adversarial regularization are more prone to compression and noise.

*H3 (temporal–spatial integration): supported*

The last hypothesis was also confirmed because the implementation of temporal modelling using Vision Transformer (ViT) + Temporal Convolutional Network (TCN) has led to a significant improvement in distortion resistance. The hybrid ViT + TCN model received an AUC of 89.3% in the degraded conditions, which would be significantly better than the ViT-only spatial model by an average of 4 AUCs. The fact that degradation was reduced (Δ = −8.6 vs. −12.3) confirms that temporal consistency modelling can be used to conserve inter-frame consistency and reduce artefact suppression. The amount of the computational overhead borne by the improvement was relatively small (about 20 percent for the increase in inference time), but the benefit in robustness is worth the tradeoff to be deployed in high-stakes forensic systems.

### 4.3 Discussion of findings

This research paper has shown that there is a significant amount of performance variation across the existing state-of-the-art face-swap detection algorithms in the face of actual distortions in the real world, such as JPEG compression, Gaussian noise, low-light gamma-correction, and motion blur. In line with H1, significant degradation in model detectors and AUC scores at distortion levels in all models, and this is one of the key points that proves that robustness is a primary constraint of present deepfake detectors. It is worth noting that FSD-GAN and FakeTracer worked better across all forms of distortion, and both FaceForensics++, Celeb-DF v2, and DeeperForensics-1.0, with over 80% accuracy, even at high levels of degradation. This result endorses H2 and goes in line with Sun et al. (2025) and Mishkhal et al. (2025), who suggested that models that entail the implementation of such properties as generator-specific traces or latent noise fingerprints may withstand compression and noise perturbations better than regular CNNs. The strength of the aforementioned GAN detectors shows the importance of adversarial learning in the latent space when maintaining discriminative information in degraded visual situations.

These differences were found to be statistically significant using the ANOVA and Tukey HSD tests [*F* (4, 60) = 18.27, *p <* 0.001; *η*^2^ = 0.55]. The pair-wise tests revealed that FSD-GAN and FakeTracer are much more robust compared to MesoNet and XceptionNet (*p <* 0.01), whereas Hybrid + Landmark was medium-strong (*p <* 0.05). These results build on the results of T et al. (2024) and Weerawardana and Fernando (2021), indicating that lightweight CNNs, despite being effective, do not have adequate generalization in low-quality or adversarial settings. The trade-off between accuracy and computational efficiency is a performance issue that has posed a design challenge, especially in real-time or embedded designs that are forced to work within constrained processing protocols. The findings show that the strength of robustness cannot be determined by inference with the large clean-data accuracy, but rather by the validation with the distortion awareness to be practical.

XceptionNet did not have such a solid performance in motion-blur settings and in low-illumination settings, despite having a solid performance on clean datasets. The susceptibility of this lies in its need for a high-frequency space feature that is killed easily by compression and blur. This fact matches with Waseem et al. (2023) and Zhang et al. (2021), who concluded that deep CNNs that are trained on uniform benchmarks tend to overfit to texture artefacts of the dataset. Therefore, the robustness can be enhanced with the help of cross-domain fine-tuning and cross-domain augmentation-rich training that subjects the network to distortions in real life. In their absence, even deep architectures that have a large number of parameters will not be reliable when deployed into production environments, including social media video streams.

A sensible trade-off was seen with the Hybrid + Landmark detector, which traded off between the speed of inference and moderate robustness. It could obtain *Δ*-AUC = −22.2 by using geometry facial features with shallow convolutional features, and was better than lightweight CNNs and at the same time more efficient than models based on GAN. This confirms the results of Nguyen-Nhat et al. (2023), Jawad et al. (2024), and Zhao et al. (2025) that the hybrid geometry-appearance models provide an important accuracy-efficiency trade-off that can be applied on mobile and embedded devices. The outcome is also acclimatized with Hriez (2025), who suggested contour-hybrid watermarking and landmark-based analysis as proactive attacks against face-swap attacks.

FaceForensics++, Celeb-DF v2, and DeeperForensics-1.0, which are used in cross-dataset experiments, display strong domain-shift effects, which validate the earlier statements of Tian et al. (2021) and Lyu et al. (2019). In cross-dataset evaluation models trained on one dataset were evaluated on another, AUC reduced by 1,527 percent, and it is indicative of how dataset bias and the lack of environmental variety overstate reported results on managed benchmarks. These results support the arguments that cross-benchmarking and distortion-conscious training pipelines need to be pursued that are more representative of the reality of digital media (Xia et al., 2025). This study adds a contribution to a more ecologically valid evaluation of generalization in models because it uses DeeperForensics-1.0, which adds differences in lighting, compression, and camera sensors.

Lastly, testing of the ViT + TCN dual-branch model proved that H3 is correct, i.e., that the presence of temporal coherence significantly reflects robustness. The significance of the dual-branch architecture that we introduce in this study from a comparative approach to a methodological advancement. This is because it considers distortion-caused damage of features through the temporal and spatial integration aspect. The hybrid model had the least degradation rate (Δ = −8.6 p.m.) over the spatial-only ViT (Δ = −12.3 p.m.), which validates the claim that temporal feature aggregation negates artefact suppression and frame-order effects (Akhtar et al., 2024). The results are consistent with previous studies by Sabir et al. (2019) and Guera and Delp (2018), indicating that the use of temporal regularization to enhance the resilience to noise and compression is not prohibited by the latency overheads (Li et al., 2023). Collectively, these findings support all three hypotheses (H1, H2, H3) and serve as an addition to the body of knowledge about the use of robustness, domain generalization, and temporal–spatial modelling in the next-generation deepfake detection systems (Van et al., 2017; van Gerven and Bohte, 2017).

### 4.4 Strengths, limitations, and validity analysis

This study exhibits several significant advantages. Firstly, this study conducts a thorough assessment of face swap detection systems in realistic conditions where face images have been distorted by compression, noises, motion blur, and dim illumination. Secondly, the use of various benchmark databases makes the results more generalizable and unbiased due to avoiding any particular data source. Thirdly, applying various statistical methods, such as ANOVA and Tukey’s HSD test, increases the robustness of the results and ensures scientific validity.

Despite strong empirical support, several threats to validity must be acknowledged.

- Domain shift: even though three datasets were used, unseen real-world manipulations or camera pipelines could introduce unseen artifacts that differ from benchmark domains. Future research should employ continual domain adaptation and open-set testing to validate performance under uncontrolled conditions.
- Tuning fairness: although all models were trained under identical budgets, subtle differences in architecture depth and parameter counts may have influenced convergence speed and regularization behavior. Cross-architecture hyperparameter optimization or automated tuning (e.g., Bayesian search) could mitigate this potential imbalance.
- Overfitting: fine-tuning on *FaceForensics++* might bias detectors toward synthetic patterns specific to that dataset. The inclusion of diverse, uncurated internet samples or synthetic augmentations should be explored to reduce overfitting to benchmark artifacts.
- Training protocol variability: the evaluated models follow different training strategies, including pretrained, fine-tuned, and trained-from-scratch configurations. While this reflects their original design and ensures realistic benchmarking aligned with prior literature, it introduces variability in initialization and learned feature representations. Such differences may influence convergence behavior and robustness outcomes, potentially affecting strict comparability across models. Future work should explore unified training protocols or controlled re-training to further standardize evaluation.
- Qualitative failure analysis: qualitative inspection of failure cases reveals that most CNN-based models (e.g., XceptionNet, MesoNet) misclassify heavily compressed or blurred samples due to loss of fine-grained facial artifacts. Under low-light conditions, models often fail to capture subtle texture inconsistencies, leading to increased false negatives. GAN-based models (FSD-GAN, FakeTracer) show better resilience but occasionally confuse real videos with strong compression artifacts as fake. The proposed ViT + TCN model demonstrates improved stability by leveraging temporal consistency, though minor failures occur in extremely noisy sequences with inconsistent frame quality.

The experiment design guarantees the validity of the study because all data splits, preprocessors, and model training conditions are uniform for all models; at the same time, the usage of different measures of effectiveness, including accuracy, F1, AUC, and inference time, makes it possible to provide an extensive analysis of the model’s performance, while the statistical methods applied confirm the significance of the differences found; besides, the findings obtained are consistent with empirical data and previous research because the identified trends can be explained by the inherent vulnerabilities of CNN-based fingerprint detectors under distortions, whereas the robustness of GAN-based detectors suggests that the concept of latent fingerprints is valid; finally, the better results provided by the ViT + TCN approach are evidence of the importance of temporal consistency in reducing the impact of image distortions, thus providing logical conclusions based on the obtained results.

From a deployment perspective, practical applications must balance accuracy, latency, and compute cost.

- In high-security or forensic verification contexts (e.g., law enforcement, digital-evidence screening), maximizing accuracy and robustness is paramount; hence, FSD-GAN or FakeTracer should be prioritized despite higher inference latency (~11–12 ms / frame).
- For mobile or embedded systems such as social-media moderation or camera-level authentication, Hybrid + Landmark or MesoNet may be preferred, offering near-real-time throughput with moderate accuracy.
- In cloud or batch-processing pipelines, ViT + TCN provides an effective compromise, achieving superior robustness (*Δ* ≤ −9%) while remaining computationally feasible on GPU clusters.

Hence, practitioners should select architectures based on operational constraints, integrating distortion-aware retraining, cross-dataset validation, and temporal modeling to ensure sustained performance across diverse real-world deployment environments.

The study demonstrates strong methodological rigor through controlled experimental design, cross-dataset evaluation, and statistically validated comparisons. The results align with established findings in the literature, supporting the reliability of the interpretations. While limitations such as dataset bias, potential overfitting, and restricted distortion diversity may affect generalizability, they do not undermine the core conclusions. The analysis provides a valid and meaningful contribution toward understanding the robustness of face-swap detection models in real-world conditions.

## 5 Conclusion

This study systematically evaluated the robustness of five leading face-swap detection models, XceptionNet, MesoNet, FSD-GAN, FakeTracer, and Hybrid + Landmark across three benchmark datasets (FaceForensics++, Celeb-DF v2, and DeeperForensics-1.0) and multiple distortion types, including JPEG compression, Gaussian noise, motion blur, and low-light gamma adjustment.

The experimental results confirmed that:

- All models experienced performance degradation under distortion, validating H1 (Distortion Sensitivity).
- GAN-based detectors (FSD-GAN, FakeTracer) demonstrated the highest resilience and lowest degradation rates, supporting H2 (GAN-Based Robustness).
- The proposed ViT + TCN temporal–spatial hybrid achieved the best robustness–efficiency trade-off, confirming H3 (Temporal–Spatial Integration).
- ANOVA and Tukey *post-hoc* analyses confirmed significant architectural effects (*p <* 0.001, *η*^2^ = 0.55), while correlation results (*r* = −0.83) indicated strong sensitivity to distortion levels.

The findings highlight that while state-of-the-art deepfake detectors achieve excellent accuracy under controlled conditions, their real-world robustness remains limited by distortion, dataset bias, and computational trade-offs. The integration of adversarial latent learning and temporal coherence offers a promising path toward more resilient, deployment-ready detection systems.

The proposed ViT + TCN dual-branch framework represents a central methodological contribution of this study rather than a supplementary experimental component. By explicitly integrating spatial and temporal representations, the model directly addresses the observed limitations of existing detectors under distortion, providing a practical and scalable solution for real-world deepfake detection scenarios. This reinforces the study’s contribution beyond benchmarking, positioning it as both an evaluative and architectural advancement.

### 5.1 Recommendations for practitioners

To translate these insights into actionable design and deployment guidance, the following recommendations are provided:

*Model selection by use case and constraint*

- Under heavy compression or poor video quality.
- Prefer FSD-GAN or FakeTracer, which retained >80% accuracy and the lowest Δ-AUC (≈ − 15%).
- These models are best for forensic, legal, or government applications requiring high reliability over speed.
- On mobile or embedded devices (limited compute):
- Deploy Hybrid + Landmark, which balances detection accuracy (≈78%) with low latency and memory efficiency.
- Ideal for real-time content moderation or edge-based camera validation.
- In cloud or batch processing environments:
- Adopt ViT + TCN (Temporal–Spatial Hybrid) for scalable forensic pipelines, achieving Δ ≤ −9% robustness with acceptable GPU inference cost.
- Suitable for platform-level monitoring, archival screening, and API-based verification systems.
- For quick client-side screening or social-media flagging:
- MesoNet offers minimal latency (≈4 ms/frame) but should be combined with distortion-aware retraining to reduce false negatives.
- In environments emphasizing visual clarity (e.g., broadcast media):
- XceptionNet performs strongly on clean datasets but degrades sharply under blur and noise; it is best used after pre-filtering or re-encoding pipelines.

*Training and evaluation guidance*

- Employ cross-dataset fine-tuning (e.g., FF++ → Celeb-DF → DeeperForensics) to mitigate domain shift and enhance generalization.
- Integrate distortion simulation (compression, blur, noise, gamma correction) into the training pipeline to improve real-world performance.
- Use balanced hyperparameter tuning to ensure fairness across architectures with different depths and parameter counts.
- Avoid overfitting by including unseen manipulations and camera variations in validation splits.

*Deployment and performance optimization*

- For accuracy-critical systems, prioritize GAN-based models and allocate sufficient GPU resources to maintain inference stability.
- For low-latency deployments, use lightweight or hybrid models with quantization or pruning optimizations.
- Consider a two-stage approach:

   ◦ Fast pre-screening with MesoNet or Hybrid + Landmark.
   ◦ Deep forensic verification using FSD-GAN or ViT + TCN.

- Monitor energy consumption and latency trade-offs during deployment; FSD-GAN averaged ~12 ms/frame vs. Hybrid + Landmark’s ~ 6 ms/frame on RTX A6000.

*Future development and policy implications*

- Encourage standardized cross-condition benchmarks that include varied compression levels, lighting, and real-world artifacts.
- Integrate explainability modules (e.g., saliency maps, artifact localization) to improve model interpretability for forensic use.
- Promote transparent data and code sharing, as planned in this study, to enable replicability and comparative robustness evaluation.

### 5.2 Future research directions

Although this research paper presents a thorough assessment of the face- swap defectiveness in the presence of various distortions and datasets, a number of valuable research opportunities arise that should be pursued to develop further theory and practice in the field of deep fake-forensics.

1 *Cross-domain generalization and continuous adaptation*

Future studies aiming at creating domain-adaptive and lifelong learning approaches, which allow detection models to be maintained in unseen environments, should be performed. The existing detectors being resistant to certain distortions are vulnerable to collapse in performance in the case of novel data distributions or pipelines of syndrome synthesis. The use of meta-learning and unsupervised domain adaptation and federated model updating techniques might allow detectors to be able to miniplate themselves to new data sources without a long period of retraining. In addition, open-set deepfake detection is to be considered more, whereby the models are capable of identifying new types of manipulation not detected during the training.

2 *Multimodal and temporal fusion for authenticity verification*

Further systems ought to consider the potential to combine multimodal visual, audio, and physiological signals (e.g., blink rate, pulse signal, or speech–lip synchronization buildings) on top of the already proven strengths of the ViT + TCN hybrid. By jointly using spatial–temporal visual features with cross-modal consistency checks, significant improvements in resilience to the advanced multimodal deepfakes can be achieved. Future architectures, including Vision Language Transformers, Audiovisual Graph Networks, might also form the basis of holistic content authenticity evaluation about various manipulation methods.

3 *Distortion-aware and ecologically valid datasets*

The construction of ecologically valid, cross-platform datasets, which are able to capture real-world post-processing effects of social media recompression, transmission noise, variable frame rates, and device-level differences, is required to reduce dataset bias in the future. Similar efforts as DeeperForensics-1.0 must be extended to crowd-sourced or stream information so that models can learn pattern-oriented invariant forensics. The multi-condition evaluation protocols used in benchmarking standards must demand test models to be subjected to multi-condition distortions to obtain reproducible and practical candidates.

4 *Energy-efficient and sustainable model deployment*

Since deep fake detectors are expensive in terms of computation, particularly GAN-based and transformer-based convolutional neural networks, future research can explore energy-efficient methods such as model pruning, quantization, and knowledge distillation. Frameworks that are edge-compatible and optimization of inference that consume low power will be important to scaling their detection tool to smartphones, IoT cameras, and network edge devices. Reporting on energy profiling is also advised with accuracy metrics in order to promote sustainability and responsible development of AI.

5 *Explainability, trust, and forensic governance*

Lastly, the application of deep fake detection in legal, journalistic, or security spheres of the real world requires explainable AI (XAI) and visible governance frameworks. Future models are to include artefact attribution maps, decision rationale visualizations, and traceability logs to make them easier to interpret and provide legal admissibility. It must also be ensured that the policymakers and regulators of these platforms build standardized certification procedures of detection tools to maintain ethical use, compliance with data privacy, as well as forensic reliability.

## Data Availability

The original contributions presented in the study are included in the article/supplementary material, further inquiries can be directed to the corresponding author.

## Footnotes

1
https://github.com/deepfakestudy/robustness2025
