# Correction: Evaluating the real-world robustness of face-swap detection models under compression and noise

**DOI:** 10.3389/frai.2026.1978866

**Published:** 2026-09-11

**Authors:** Baocai Li, Fazli Bin Azzali, Nik Fatinah binti N. Mohd Farid

**Affiliations:** School of Computing, Universiti Utara Malaysia, Sintok, Malaysia

**Keywords:** compression artifacts, Deepfake, face-swap detection, FSD-GAN, media distortion, real-world evaluation, robustness, XceptionNet

Author Baocai Li was incorrectly published as Li Baocai. The correct bibliographic citation is Li B.

The original version of this article has been updated.

